# Preservation of gallic acid and methyl gallate on purified Kilka fish oil oxidation by Rancimat

**DOI:** 10.1002/fsn3.1264

**Published:** 2019-11-14

**Authors:** Maryam Asnaashari, Reza Farhoosh, Reza Farahmandfar

**Affiliations:** ^1^ Department of Food Science and Technology Sari Agricultural Sciences and Natural Resources University (SANRU) Sari Iran; ^2^ Department of Food Science and Technology Ferdowsi University of Mashhad (FUM) Mashhad Iran

**Keywords:** antioxidant activity, gallic acid, kilka oil, kinetic, methyl gallate, rancimat

## Abstract

Kinetic analysis of gallic acid and methyl gallate in purified Kilka oil was studied in the concentration, range 200–1600 ppm, during autooxidation in Rancimat test at 60°C. The stabilization factor (F), the oxidation rate ratio (ORR), the activity (A), and the mean rate of antioxidant consumption (W_inh_) were determined. The scavenging activity of 1,1‐diphenyl‐2‐picrylhydrazyl (DPPH) radicals decreased in the order gallic acid > methyl gallate > α‐tocopherol > BHT as observed in Kilka oil. There were no significant differences between the effectiveness and strength and activity of gallic acid and methyl gallate at concentrations 200, 400, and 800 ppm. However, above 800 ppm, the activity of methyl gallate became higher than gallic acid, because methyl gallate despite gallic acid did not participate in any chain propagation reactions.

## Graphical abstract

Lipid oxidation is one of the most food deterioration cause and reduces edible oil quality. Antioxidants as food additives are used to enhance oils shelf life. Antioxidant addition into the edible oil leads to change in process kinetics. Phenolic acids such as gallic acid and methyl gallate have various abilities to act as antioxidants in food products and change the lipid oxidation mechanism in noninhibited and inhibited kinetic regime. Although the effect of phenolic acids on different lipid systems have been confirmed in the numerous researches, there is no kinetic research about the mechanism of gallic acid and methyl gallate on lipid oxidation process. Thus, the accurate knowledge on the oxidation kinetics of these phenolic acids is necessary to predict these antioxidant behaviors in different lipid systems during storage.

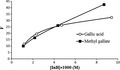

## 1 INTRODUCTION

Long‐chain polyunsaturated fatty acids (PUFAs) are identified as healthiness compounds observed in marine oil. The marine fish oils are renowned sources of PUFAs, namely eicosapentaenoic acid (EPA) and docosahexaenoic acid (DHA), which were health beneficial and potential for preventing diseases such as cancer and immune disorders. Besides, omega‐3 polyunsaturated fatty acids of fish oil are essential in brain and muscle development and lower plasma triglyceride levels (Horrocks & Yeo, 1999; Uauy & Valenzuela, 2000). On the other hand, highly unsaturated fish oils are notably prone to oxidation and produce unlikeable rancid smells and off‐flavors (Jacobsen, Adler‐Nissen, & Meyer, 1999).

In the food industry, some food deterioration, nutritional losses, and unpleasant flavors occur during lipid oxidation. The distasteful changes in edible oils increase when reacting with oxygen. Oxidative rancidity cannot be stopped by reducing the storage temperature because it is a chemical reaction with low activation energy (Asnaashari, Tajik, & Khodaparast, 2015; Farhoosh, Johnny, Asnaashari, Molaahmadibahraseman, & Sharif, 2016). Antioxidants are utilized as food ingredient to prolong the shelf life of oils during frying and storage. Using antioxidants are evaluated by different factors including effectiveness and legislation cost (Asnaashari, Hashemi, Mehr, & Asadi Yousefabad, 2015; Sun, Wang, Chen, & Li, 2011). Both natural and synthetic antioxidants have been developed to preclude rancidity and undesirable flavor in fish oil (Horn, Nielsen, & Jacobsen, 2009). Due to positive effects of natural antioxidants, as a consequence of the different side effects of synthetic antioxidants, the consumer demands have extraordinarily improved (Asnaashari, Asnaashari, Ehtiati, & Farahmandfar, 2015).

Gallic acid as a natural phenolic compound and its esters like methyl, octyl, and propyl gallates are broadly utilized in cosmetic, pharmaceutical, and food manufacturing. Gallic acid is originated in variety of plants comprising strawberries, barberry, mango peels, leaves, and walnut (Asnaashari, Farhoosh, & Sharif, 2014). Gallic acid accomplishes as an effective antioxidant and facilitates to protect our cells contrary to oxidation and preclude heart disease (Farhoosh, Sharif, Asnaashari, Johnny, & Molaahmadibahraseman, 2016; Polewski, Kniat, & Slawinska, 2002). Although the antioxidative properties of the phenolic acids were considered in the numerous researches (Eshghi, Asnaashari, Haddad Khodaparast, & Hosseini, 2014; Farahmandfar, Asnaashari, & Sayyad, 2015; Thurmann & Herrmann, 1980; Yanishlieva & Marinova, 1995), there is no kinetic research in the literature about the mechanism of gallic acid and methyl gallate.

The objective of this kinetic study was to obtain fundamental information about antioxidative behavior of gallic acid and methyl gallate at 60°C in the Rancimat test at various concentration levels (200, 400, 800, and 1,600 ppm). Furthermore, the activity and the mechanism of these two antioxidants in the triacylglycerols of Kilka oil have been discussed.

## 2 MATERIALS AND METHODS

### 2.1 Materials

**Figure 1:**
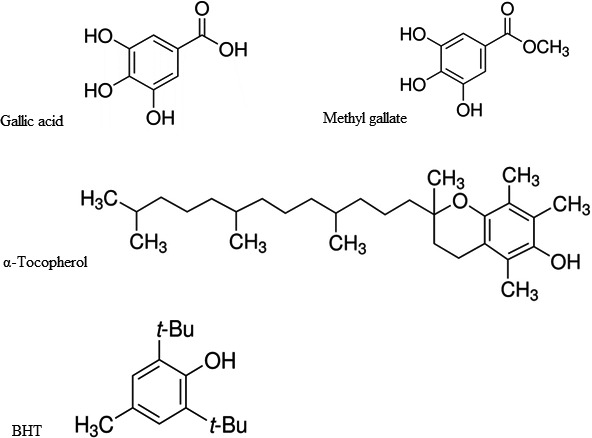
Molecular structure of gallic acid, methyl gallate, and α‐tocopherol and BHT

No added components of crude Kilka oil with distinct physicochemical properties (Table 1) were provided from Parskilka Company. Antioxidants like gallic acid, α‐tocopherol, BHT, and methyl gallate were purchased from Merck and Sigma Chemical Companies. The structural formulas of these antioxidants are shown in Figure 1. The solvents and chemicals were as analytical reagent grade and obtained from Sigma and Merck Chemical Companies.

**Table 1:** Physicochemical characteristics of the crude Kilka oil^a^

Parameter	Amount
Wax content (%)	11.07 ± 0.68
Unsaphonifable matter (%)	2.60 ± 0.22
Sterol content (%)	7.523 ± 0.28
Total tocopherols content (mg α‐tocopherol per kg oil)	2.89 ± 4.89
Total phenolic content (mg gallic acid per kg oil)	134.02 ± 6.61
Saponification number (mg KOH per g oil)	53.72 ± 3.54
Peroxide value (meq O_2_ per kg oil)	1.78 ± 0.04
Acid value (mg KOH per g oil)	13.98 ± 0.43
Density (g/cm^3^)	950.72 ± 0.52
Total polar compounds content (%)	52.73 ± 0.92

a Mean ± *SD* (standard deviation) of triplicate determinations.

### 2.2 Purification of the Kilka oil

Kilka oil (120 g) was purified chromatographically to eliminate natural antioxidants. Three absorbents consist of 60 g aluminum oxide 60 (activated at 200°C for 3 hr, neutral) in bottom of the column, 80 g silica gel (activated at 160°C for 3 hr, 60–200 mesh), and 2 g carbon active on the top were used in glass column (50 × 5 cm i.d.) sequentially. The collection vessels and chromatographic column were covered by aluminum foil, and the suction (without solvent) draw the oil through the column.

### 2.3 Rancimat test

Methyl gallate and gallic acid at different concentration of 200, 400, 800, and 1,600 ppm and also α‐tocopherol and BHT (200 ppm) were separately added to purified Kilka oil and then subjected to the 743 Rancimat apparatus from Metrohm at 60°C. Sample size and airflow rate were 3 g and 15 L/s, respectively. Electrodes, measuring vessels, connecting tubes, and glassware were cleaned before the assay.

### 2.4 Determination of IP of oil samples

The oxidation stability of Kilka oil samples containing antioxidant was evaluated by the Rancimat instrument. In this technique, the tertiary products of oil oxidation, principally formic acid (C_1_), acetic acid (C_2_), and propionic acid (C_3_), formed under accelerated conditions were identified by continuously measuring the water electrical conductivity over time. The Rancimat test expressed the induction period (IP) as the time before rapid deterioration of the oil occurs.

The antioxidant effects on lipid oxidation of Kilka oil are discussed by kinetic parameters: Stabilization factor (F) points to the probability of chain termination of free radicals, especially peroxide radicals. Stabilization factor (F) has determined by the following formulation:

(1) $$F={\text{IP}}_{\text{inh}}/{\text{IP}}_{0}$$

where IP_inh_ and IP_0_ are induction period in the presence and absence of antioxidant. The oxidation rate ratio (ORR) is a quantity of strength, which could be calculated by following equation:

(2) $$\text{ORR}=W_{\text{inh}}/W_{0}$$

where W_inh_ and W_0_ are the slopes of the linear initiation stage of lipid oxidation in attendance of an antioxidant and control, respectively.

Activity (A), shown as follows, is a common parameter, which simultaneously indicates the efficacy of an antioxidant in termination of the oxidation process and its capability to decline the oxidation rate during the induction period.

(3) A=F/ORR

The mean rate of inhibitor consumption **(**W_inH_) determined by the formula:

(4) $$W_{\text{inH}}={\left[\text{inH}\right]}_{0}/{\text{IP}}_{\text{inh}}\;\left({\text{Ms}}^{-1}\right)$$

where [inH]_0_ and IP_inh_ are the initial concentration of antioxidant (M) and the length of induction period (s), respectively (Marinova & Yanishlieva, 1992).

### 2.5 Determination of radical scavenging activity

Total antioxidant ability was determined by DPPH assay. In the methanol solution, the samples were reacted with the stable DPPH**˙** radicals. After 30 min incubation period (25°C, dark situation), the absorbance of the solution was quantified (at 517 nm) by a spectrophotometer. DPPH free radical inhibition (I%) was calculated in the following way (Lima, Fernandes‐Ferreira, & Pereira‐Wilson, 2006):

(5) $$I\%=\left(A_{\text{blank}}-A_{\text{sample}}\right)/A_{\text{blank}}\times 100$$

where A_blank_ and A_sample_ are the absorption of control and sample solutions, respectively. The IC_50_ value, the concentration of the sample required to inhibit 50% of DPPH radical, was achieved by the linear fitted line of dose–response curve plotting between inhibition and concentrations. Antiradical power (ARP) is inverse of IC_50_, and a relative ARP is the activity of experimented compounds in relation to the strongest scavenger in which the ARP was stated as 100%.

### 2.6 Statistical analysis

The obtained data were subjected to analysis of variance by SAS software. Significant differences between means were determined by Duncan's multiple range tests at the 5% probability level (*p* < .05).

## 3 RESULTS AND DISCUSSION

### 3.1 Scavenging effect on DPPH radicals

**Figure 2:**
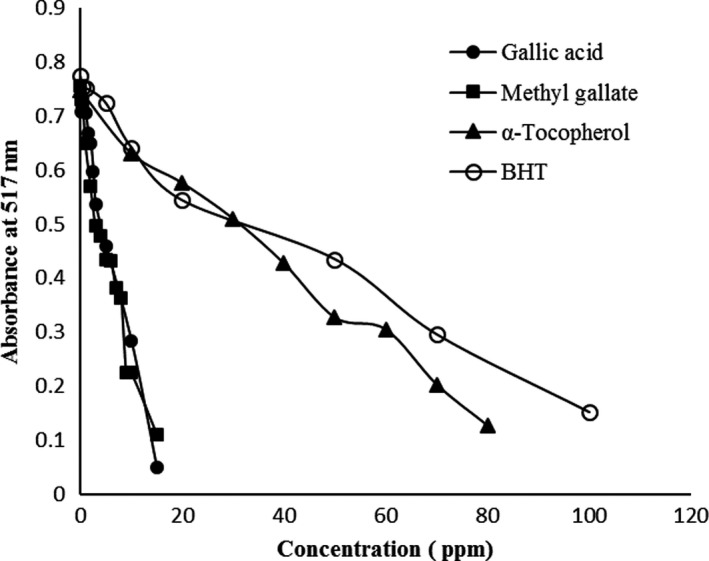
Radical scavenging activities of gallic acid and methyl gallate, α‐tocopherol, and BHT on DPPH, as measured by changes in absorbance at 517 nm

DPPH free radical scavenging by antioxidants is due to their hydrogen denoting ability. The dose–response plotting curve between absorbance and concentrations for the free radical scavenging capacity of BHT, α‐tocopherol, methyl gallate, and gallic acid by the DPPH coloring test were presented in Figure 2. The absorbance of samples reduced severely with the increase in the concentration of antioxidant to a certain extent and then diminished slightly with further concentrations. At a dosage of 20 mg/ml of compounds, the highest DPPH scavenging ability belonged to gallic acid, which is found to be 97.6% and followed by methyl gallate (85.15%), BHT (29.71%), and α‐tocopherol (22.89%). While in 50 mg/ml dosage, the order of DPPH scavenging ability of samples was slightly different because the scavenging ability of α‐tocopherol with 56.22% was higher than BHT with 43.79%. Indeed, the various radical scavenging observed can be associated with different abilities of individual phenolic acids to react with DPPH to give a stable nonradical product.

Values for half‐inhibition concentration (IC_50_), antiradical power (ARP), and a relative ARP of experimented compounds are showed in Table 2. The antiradical activity drops off in the order gallic acid > methyl gallate > α‐tocopherol > BHT. Actually, gallic acid required lower doses than methyl gallate, α‐tocopherol, and BHT to reach 50% DPPH***˙*** scavenging ability. The high value of scavenging activity for gallic acid was reported by (Karamac, Kosiñska, & Pegg, 2005). In the phenolic acids, hydroxyl groups attached to the benzoic molecule are important for free radical scavenging efficiency. As can be seen in Table 2, gallic acid, with four hydroxyl groups, was the strongest antioxidant. Researchers showed that methylation of OH group diminished ARP value in methyl gallate (Kikuzaki, Hisamoto, Hirose, Akiyama, & Taniguchi, 2002).

**Table 2:** Antiradical activities of selected phenolic acids against DPPH^a^

Compound	IC_50_	ARP	Relative ARP
Gallic acid	5.006	0.2	100
Methyl gallate	7	0.142	71.514
α‐Tocopherol	45.35	0.022	11.039
BHT	50.003	0.019	10.011

a Mean ± *SD* (standard deviation) of triplicate determinations.

### 3.2 Kinetic study of gallic acid and methyl gallate in triacylglycerols of Kilka oil

Fatty acids composition of the Kilka fish oil is shown in Table 3. This oil was predominantly constituted of oleic, palmitic, palmitoleic, linoleic, myristic, eicosapentaenoic, and docosahexaenoic acids. Among the fatty acids, the maximum amount of the polyunsaturated, monounsaturated, and saturated fatty acids were linoleic acid (8.16%), oleic acid (27.51%), and palmitic acid (17.31%), respectively. The fatty acids composition of Kilka oil showed that the amount of EPA (6.35%) and DHA (5.89%) was marvelous. The fatty acids composition of the Kilka oil used in this study was by data reported in the literature (Frankel, Satué‐Gracia, Meyer, & German, 2002). Furthermore, (EPA + DHA)/C16:0 ratio was 0.71 and the total content of PUFA and ω‐3 fatty acids were 21.77 and 13.40%, respectively. The remarkable content of these fatty acids make Kilka oil as an important functional food. However, due to its high susceptibility to oxidation, antioxidants need to be added.

**Table 3:** Fatty acid composition of purified Kilka oil

Fatty acid	Percentage (%)
C14:0	6.22 ± 0.05
C16:0	17.31 ± 0.01
C16:1	13.23 ± 0.07
C17:0	1.89 ± 0.06
C18:0	3.23 ± 0.04
C18:1	27.51 ± 0.06
C18:2	8.16 ± 0.15
C18:3	1.17 ± 0.09
C20:0	1.16 ± 0.06
C20:4	0.21 ± 0.03
C20:5 (EPA)	6.35 ± 0.05
C22:6 (DHA)	5.89 ± 0.03
SFA	29.80 ± 0.12
MUFA	40.74 ± 0.01
ω_3_	13.40 ± 0.11
PUFA	21.77 ± 0.06
PUFA/SFA	0.73 ± 0.01
(EPA + DHA)/C16:0	0.71 ± 0.002
IV [g I_2_/100 g oil]	114.99 ± 1.10

**Figure 3:**
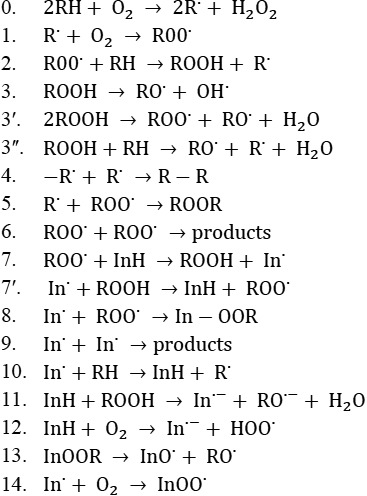
Noninhibited (0–6) and inhibited (7–14) oxidation reaction. InH, inhibitor; RH, oxidation lipid substrate; ROO^.^, peroxide radical.

Mechanism of lipid oxidation in a noninhibited and inhibited kinetic regime (a sufficiently high oxygen concentration) is presented in Figure 3. Adding an antioxidant into the lipid system causes modification in the process kinetics. In this study, the effect of the gallic acid, methyl gallate, α‐tocopherol, and BHT in triacylglycerol's of Kilka oil relies on their molecules and their result radicals in a series of reactions (Scheme 1). In the following schemes, LH is the oxidizing lipid substrate, InH is the inhibitor, in˙ is the radical of antioxidant, and LOO˙ is the peroxide radical.

The attained parameters for gallic acid and methyl gallate (200, 400, 800, and 1,600 ppm) and 200 ppm of α‐tocopherol and BHT, after processing the kinetic curves, are given in Table 4. It can be seen that as the concentration of antioxidants raised, the efficacy enhanced because of chain termination of free radicals using interaction with peroxide radicals (reaction 7). The strength of antioxidants which specifies the chance of antioxidant moieties participating in other reactions, for example 7, 10, 11, 12, and 14, also increased in higher concentrations. Indeed, the possibility of occurring reactions 7–14 relies on the antioxidant structure, temperature, lipid medium, and other conditions of the oxidation process (Marinova & Yanishlieva, 2003). As shown in Table 3, the effectiveness, strength, and activity of gallic acid and methyl gallate were not significantly different, except 1,600 ppm of methyl gallate, which was remarkably higher than gallic acid. The activity (A parameter) of 200 ppm of experimented compounds decreased in the following order: gallic acid ~ methyl gallate > α‐tocopherol > BHT. By comparing the order of antioxidants activity in oil with the DPPH radical scavenging activity, it is obvious that methyl gallate was almost as same effective as gallic acid, despite being less polar and radical scavenging activity. Interactions between the methoxy group of methyl gallate and triglyceride molecular results in meaningful antioxidant activity in edible oil. Also, intramolecular interactions between the oxygen atom of the hydroxyl group and the hydrogen atoms of the methylene group of methyl gallate in the meta position of the gallate can increase the hydrogen donor ability and antioxidant activity of methyl gallate (Stöckmann, Schwarz, & Huynh‐Ba, 2000).

**Table 4:** Kinetic parameters characterizing the inhibited lipid oxidation of purified Kilka oil at 60°C in PV˳ = 0 meq/kg, IP˳ = 1.60 hr, W˳ = 68.1 × 10^–9^ M/S

Antioxidant	Inhibitor concentration		F^b^	ORR^c^	A^d^	W_inh_(×10^9^) (M/s)^e^	W_InH_(×10^10^)(M/s)^f^
	[InH]^a^ mg/L	[InH]×1,000(M)					
Gallic acid	200	1.18	11.53	0.38	30.38	23.5	177.32
	400	2.35	19.64	0.34	57.52	21.3	207.3
	800	4.70	26.31	0.27	97.56	16.6	309.5
	1,600	9.41	32.47	0.17	186.5	10.8	502.1
Methyl gallate	200	1.09	9.96	0.37	26.63	23.1	189.1
	400	2.17	16.57	0.30	53.58	19.1	226.9
	800	4.34	26.04	0.28	93.14	17.4	288.8
	1,600	8.69	42.54	0.21	194.6	13.5	353.9
BHT	200	0.91	7.96	0.37	21.45	23.1	197.9
α‐Tocopherol	200	0.46	6.98	0.32	24.37	13.2	105.4

a Concentration of the antioxidant.

b Stabilization factor.

c Oxidative rate ratio.

d Activity.

e Oxidation rate of inhibited oxidation (M/s).

f Mean rate of inhibitor consumption (M/s).

**Figure 4:**
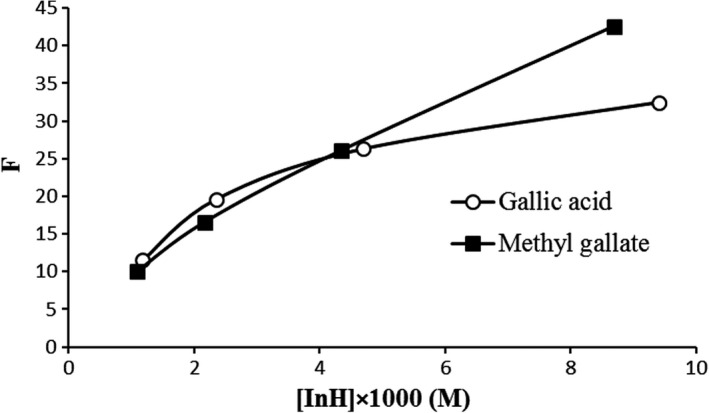
Dependence of gallic acid and methyl gallate stabilization factor F on its concentration [InH] during oxidation of Kilka oil at 60°C

Figure 4 shows dependency of the stabilization factor (F) on the concentrations of gallic acid and methyl gallate through oxidation of Kilka oil (at 60°C). As can be seen in Figure 4, in lower concentrations, the effectiveness of gallic acid was basically as same as that of methyl gallate. However, at concentrations above 4 × 1,000 M methyl gallate were more efficient as compared with gallic acid.

Nonlinear dependence of the F with gallic acid and methyl gallate concentrations is attributed to the participation of their molecules in reactions other than the main reaction of chain termination (7), namely reaction (11) and/or (12). In this case, there is a relationship between the mean rate of antioxidant consumption W_InH_ and the antioxidant concentration [InH] (Emanuel, Denisov, & Maizuss, 1965):

(6) $$W_{\ln H}=W_{i}/f+K_{\text{eff}}\;{\left[\ln H\right]}^{n}$$

where W_i_ is the mean rate of inhibition during the induction period of the inhibited oxidation (M/s), ƒ is the stoichiometric coefficient of inhibition, which determines how many radicals perish in an inhibitor molecule, and K_eff_ is the rate constant of antioxidant consumption in side reaction(s) of chain propagation (Marinova & Yanishlieva, 2003).

**Figure 5:**
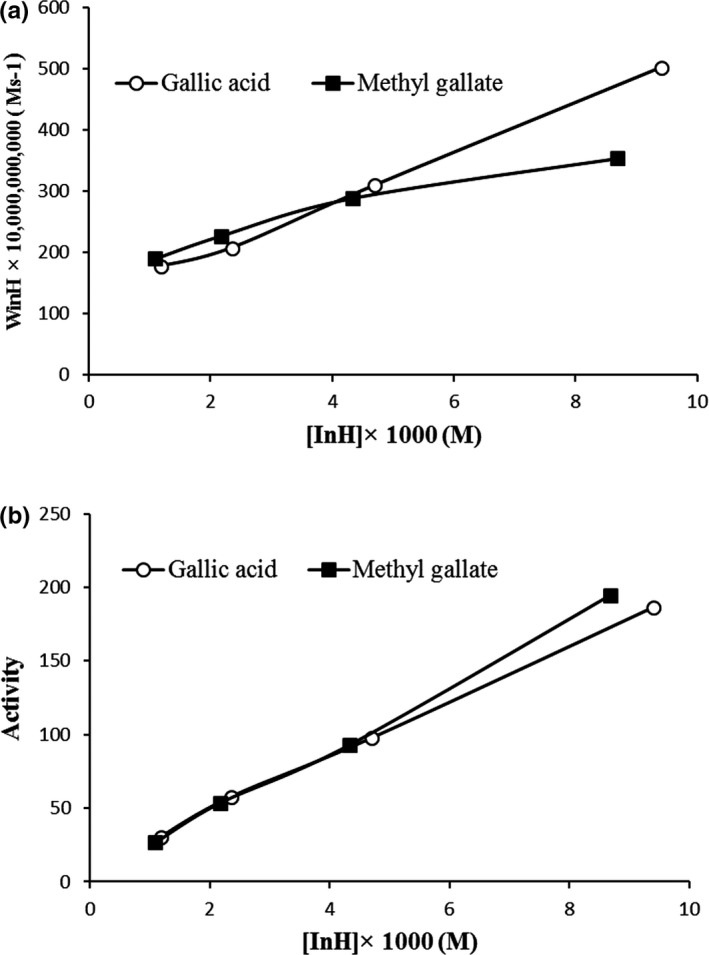
(a) Dependence of the mean rate of consumption W_inH_ of gallic acid and methyl gallate on their concentration [InH] b) Dependence of the antioxidant activity of gallic acid and methyl gallate on the concentrations during the oxidation of Kilka oil at 60°C

The dependence of W_InH_ on [InH] results for gallic acid and methyl gallate at 60°C showed linearity for *n* = 1 (Figure 5a), which means that these two antioxidants only participate in one of the two side reactions of chain propagation (11 or 12). Indeed, reaction 11 is dependent on the hydroperoxide reactivity. So, the main side reaction in which the antioxidants in inhibited lipid oxidation participate is reaction 11 (Marinova & Yanishlieva, 1996). From the slope of the dependencies in Figure 5a, the rate constants of this side reaction were 40.256 × 10^–7^ s^−1^ and 21.09 × 10^–7^ s^−1^ for gallic acid and methyl gallate, respectively. Previous investigations on the antioxidative behavior of some phenolic acids in different lipid systems indicated that K_eff_ depended in the lipid substrate. The parameter W_i_/*f* shows the participation of the antioxidant in the initiation reactions. W_i_/*f* as determined from Figure 5a by extrapolation to zero concentration of gallic acid (121/55 M/s) and methyl gallate (178.99 M/s), which indicated that methyl gallate participates in chain initiation more than gallic acid during the oxidation. This fact may be one of the reasons for the slightly lower effectiveness and strength of methyl gallate than gallic acid.

Figure 5b clarifies the changes of antioxidant activity with increasing gallic acid and methyl gallate concentrations in the oxidation process of purified Kilka oil at 60°C. As can be seen in Figure 5b, the activities of gallic acid and methyl gallate are practically the same in the concentration interval 0.001–0.006 M. However, above this concentration the activity of methyl gallate is slightly higher than gallic acid.

In order to evaluate the participation of antioxidant radical ln˙ on chain propagation reactions, the following relations developed. The previous research showed that if ln˙ does not participate in chain propagation, this relationship is valid (Marinova & Yanishlieva, 1996).

(7) $$W_{\text{inh}}∼{\left[\ln H\right]}^{-1}$$

While if ln participates in one of the chain propagation (reaction 7, 10, and 14), this relationship is observed.

(8) $$W_{\text{inh}}∼{\left[\ln H\right]}^{-0.5}$$

**Figure 6:**
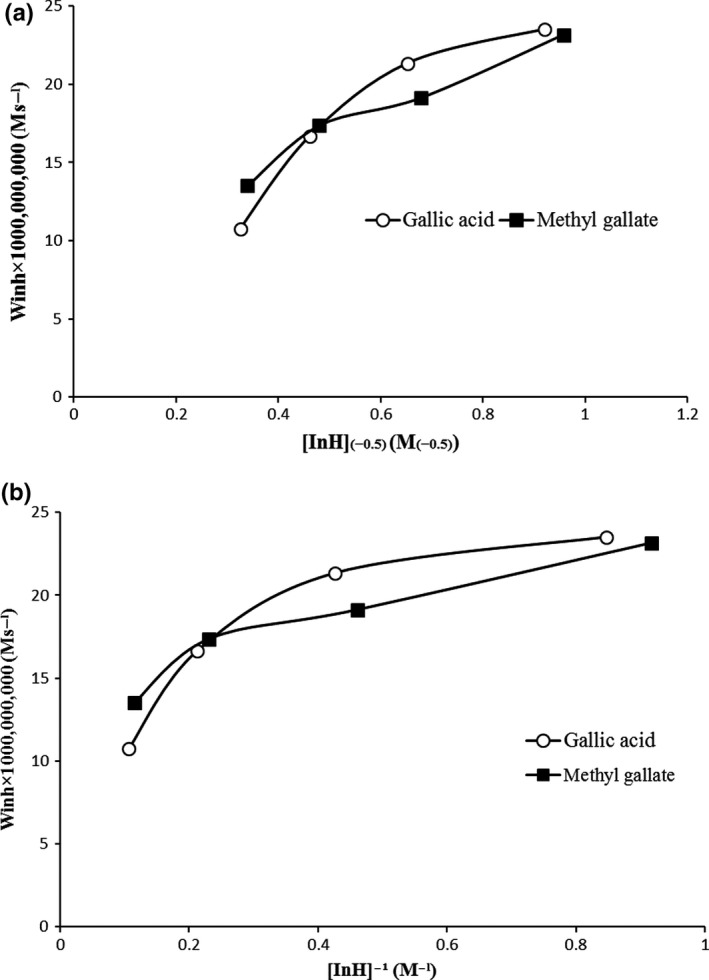
(a) Dependence of the rate of inhibited oxidation W_inh_ on the concentration of phenolic acids [InH]^−1^ (b) Dependence of the rate of inhibited oxidation W_inh_ on the concentration of phenolic acids [InH]^−0.5^ during oxidation of Kilka oil at 60°C

Processing of the results obtained from the dependence of the rate of inhibited oxidation W_inh_ on the concentrations of gallic acid and methyl gallate (7) showed the relationship was valid (Figure 6a). This means that the radicals of methyl gallate did not participate in any chain propagation. However, gallic acid indicated no linear dependence on both [InH]^−1^ and [InH]^−0.5^, which showed that the radicals of gallic acid were included in more than one reaction of chain propagation (Figure 6b). As a consequence, the stability of oil including gallic acid at 1,600 ppm is lower than oil contained methyl gallate. Moreover, based on (7) relationship for both antioxidants showed a good linear relationship at concentration lower than 800 ppm. It means that lower than 800 ppm, radicals of gallic acid and methyl gallate did not participate in chain propagation reactions. But at 1,600 ppm the mechanism became different.

## 4 CONCLUSIONS

The results of the present study indicated that methylation of benzoic acid could change the kinetic mechanism of oxidation in Kilka oil. According to the kinetic parameters obtained for gallic acid and methyl gallate, there was observed no significant differences between the activity, effectiveness, and strength of these two antioxidants at 200–800 ppm concentrations, whereas at 1,600 ppm, methyl gallate acts better than gallic acid due to no participation in any propagation chain reactions. Moreover, comparison of the activity of gallic acid and methyl gallate with α‐tocopherol and synthetic antioxidant (BHT) indicated that these two antioxidants have better potential to inhibit lipid oxidation in Kilka oil. So, they can be used as suitable alternatives to synthetic antioxidants.

## CONFLICT OF INTEREST

The authors have declared no conflict of interest.

## ETHICAL APPROVAL

This study does not involve any human or animal testing.
